# AID Enzymatic Activity Is Inversely Proportional to the Size of Cytosine C5 Orbital Cloud

**DOI:** 10.1371/journal.pone.0043279

**Published:** 2012-08-20

**Authors:** Gopinath Rangam, Kerstin-Maike Schmitz, Alexander J. A. Cobb, Svend K. Petersen-Mahrt

**Affiliations:** 1 DNA Editing in Immunity and Epigenetics, Fondazione Istituto FIRC di Oncologia Molecolare, Milano, Italy; 2 DNA Editing Lab, Clare Hall Laboratories, London Research Institute, South Mimms, United Kingdom; 3 School of Pharmacy, University of Reading, Reading, United Kingdom; National Institute on Aging, United States of America

## Abstract

Activation induced deaminase (AID) deaminates cytosine to uracil, which is required for a functional humoral immune system. Previous work demonstrated, that AID also deaminates 5-methylcytosine (5 mC). Recently, a novel vertebrate modification (5-hydroxymethylcytosine - 5 hmC) has been implicated in functioning in epigenetic reprogramming, yet no molecular pathway explaining the removal of 5 hmC has been identified. AID has been suggested to deaminate 5 hmC, with the 5 hmU product being repaired by base excision repair pathways back to cytosine. Here we demonstrate that AID’s enzymatic activity is inversely proportional to the electron cloud size of C5-cytosine - H > F > methyl >> hydroxymethyl. This makes AID an unlikely candidate to be part of 5 hmC removal.

## Introduction

Demethylation of 5-methylcytosine (5 mC) in DNA is an integral part in the maintenance of an intact epigenome and driving regulated processes like embryonic development. Due to chemical constraints DNA demethylation can only be facilitated by conversion, possibly via deaminases and oxygenases, and/or removal of the 5 mC nucleoside. Activation induced deaminase (AID) deaminates cytosine (C) or 5 mC to uracil (U) or thymine (T) [1]. Genetic ablation of AID in mice lead to a partial loss of global DNA demethylation [2], while AID seems also to be important for resetting DNA methylation marks during pluripotency formation [3] and for zebrafish development [4]. The TET protein family converts 5 mC into 5-hydroxymethylcytosine (5 hmC) [5], and although data indicate this modification can be processed further [6], [7], it is not known how the TET induced modifications lead to dC reformation. Without biochemical evidence, it was hypothesised that 5 hmC could be deaminated by AID, and in conjunction with DNA repair lead to dC formation [8], [9].

In this study we determined the precise substrate requirements for AID, by utilising our ssDNA oligonucleotide deamination assay [1] incorporating cytosine modifications (hydrogen, fluoro, CH_3_, or CH_2_OH at C5 position of dC). Our data show that while AID can deaminate unmodified cytosines or cytosine with a fluoro or a methyl group at their C5 position, 5-hydroxymethylated cytosine could not be processed by AID. Substrates with electron cloud size at C5 position of cytosine exceeding that of a methyl group are not a substrate for AID. We conclude that AID is not involved in 5 hmC conversion.

**Figure 1:**
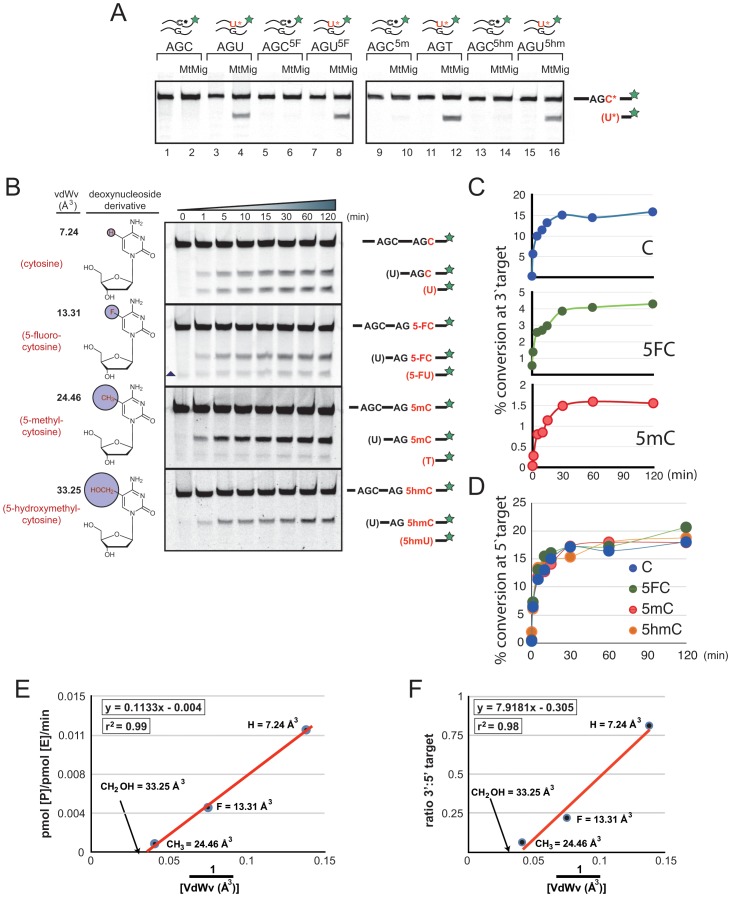
In vitro deamination of substrates with unmodified (5 ′ **AGC) and C5 modified cytosines (3**′ **AGC) by AID.** (**A**) MtMig recognizes only deaminated products of cytosine or cytosine derivatives (U, T, 5FU, 5 hmU) opposite a dG in a double stranded context. Absence of MtMig or cytosine-derivatives did not lead to product formation. (**B**) Name, vdWv of the C5, and structure of deoxynucleoside derivatives used in the assays are shown on the left of the gels. Oligos were incubated with 1.8 pmol AID for the indicated times and migration of substrate and products are indicated on the right of gels. Bases in brackets are those that have been deaminated by AID and removed by MtMig prior to cleavage. Triangle indicates a nonspecific cleavage product excluded from quantitation. One representative of three independent experiments is shown. (**C**)****Quantitation of the 3′ AGC deamination assays shown in B: 3′ AG-C -(top); 3′ AG-5FC - (middle); 3′ AG-5 mC - (bottom). Although the scales are different on the y-axis, the kinetic profiles do not significantly deviate from one another. (**D**) Quantitation of the 5′ AGC deamination of (B), indicating that the various modification at the 3′ AGC do not alter the activity of AID towards the 5′ AGC. (**E**) The average product formation in the linear phase (5 – 30 min) of the reaction from (C) was converted to pmol [P]/ pmol [E]/ min. The values were then plotted against the inverse of the vdWv from (B) for each derivative. The line of best fit and its r^2^ value are shown. The position of the theoretical value of the CH_2_OH side chain is indicated on the x-axis by an arrow. (**F**) The vdWv plotted against the average ratio of the 3′ target to that of the 5′ target for each time point. Analogous to (E), the line of best fit showed extremely high correlation (r^2^ = 0.98) and intersected the x-axis (1/25.96) at a size that is smaller than that of 5 hmC (i.e. a larger value of the inverted vdWv).

**Table 1:** Oligonucleotides used.

Figure 1A	
**SPM163**	5′-***Bt*** *-*ATTATTATTATT**AGC**TAT TTA TTTATTTATTTATTTATTT-***FITC***-3′
**SPM164**	5′-***Bt*** *-*ATTATTATTATT**AGU**TAT TTA TTTATTTATTTATTTATTT-***FITC***-3′
**HC1178**	5′-***Bt*** *-*ATTATTATTATT**AGC^5Me^**TAT TTA TTTATTTATTTATTTATTT-***FITC***-3′
**DM1854**	5′-***Bt*** *-*ATTATTATTATT**AGT**TAT TTA TTTATTTATTTATTTATTT-***FITC***-3′
**RG50**	5′-***Bt*** *-*ATTATTATTATT**AGC^5F^**TAT TTA TTTATTTATTTATTTATTT-***FITC***-3′
**RG52**	5′-***Bt*** *-*ATTATTATTATT**AGU^5F^**TAT TTA TTTATTTATTTATTTATTT-***FITC***-3′
**RG51**	5′-***Bt*** *-*ATTATTATTATT**AGC^5HM^**TAT TTA TTTATTTATTTATTTATTT-***FITC***-3′
**RG53**	5′-***Bt*** *-*ATTATTATTATT**AGU^5HM^**TAT TTA TTTATTTATTTATTTATTT-***FITC***-3′
**spm166 comp**	**5′-AAATAAATAAATAAATAAATAAATAGCTAATAATAATAAT-3′**
**Figure 1B–F** ** and ** **Fig. 2**	
**RG17**	5′-***Bt***-GTTATTGTTATTGTT**AGC**TAGT**AGC**TATTGTTATTGTTAT-***FITC***-3′
**RG22**	5′-***Bt***-GTTATTGTTATTGTT**AGC**TAGT**AGC^5Me^**TATTGTTATTGTTAT-***FITC***-3′
**RG23**	5**′**-***Bt***-GTTATTGTTATTGTT**AGC**TAGT**AGC^5F^**TATTGTTATTGTTAT-***FITC***-3′
**RG33**	5′-***Bt***-GTTATTGTTATTGTT**AGC**TAGT**AGC^5HM^**TATTGTTATTGTTAT-***FITC***-3**′**
**RG16 comp**	**5′-ATAACAATAACAATAGCTACTAGCTAACAATAACAATAAC-3′**

## Materials and Methods

Recombinant AID (HIS-tagged) was produced in *E. coli* and purified by affinity column (Ni-NTA agarose, Quiagen) as previously described [10]. The oligodeoxyribonucleotide substrates were purchased from Purimex, Germany and AltaBioscience, UK, and are described in Table 1. The ssDNA oligonucleotide deamination assay (ODA) and AID active-site titration was based on our previous protocols [1], [10], [11]. Oligos containing two cytosines in the context of two AGCs (preferred sequence target for AID), one 5′ AGC (unmodified C - serving as an internal control) and one 3′ AGC containing a modified cytosine (overview of the cytosine modifications in Figure 1B), were synthesised. For each reaction, 2.5 pmol of the 5′-biotin-tagged and 3′-fluorescein-tagged oligo was mixed with 1 ng of RNaseA in reaction buffer R (50 mM NaCl, 3 mM MgCl_2_, 40 mM KCl, 40 mM Tris HCl pH 8.0, 1 mM DTT, 10 % glycerol) in a total volume of 10 µl, denatured for 3 min at 90°C followed by immediate quenching in ice-water. Oligos were incubated with the indicated amounts of recombinant AID and for the indicated time. Percent activity of AID was determined as previously described [11], [12]. Reactions were stopped by addition of 100 µl water and denaturation for 3 min at 90°C. To recover the oligos, 8 µl of streptavidin magnetic beads (Dynal M270, Invitrogen), washed twice in TEN-M (50 mM Tris HCl pH 7.5, 10 mM EDTA, 1 M NaCl) and resuspended in fresh 750 µl TEN-M were added and oligos were allowed to bind for 15 min. Beads were collected with a magnet and washed twice in TEN-M pre-heated to 70°C and finally in TE. An excess of complementing oligo RG16 (with a G opposite the target sites) was annealed to the modified oligo in 1x MtMig reaction buffer, followed by cleavage at the site of the mismatch with MtMig (*Methanobacterium thermoautotrophicum* mismatch glycosylase; also known as thermostable TDG from Trevigen, USA catalog No 4070–500-EB) for 1 h at 47°C. Although classified as a TDG, MtMig belongs into another family of DNA glycosylases and is able to recognize various mismatches opposite a G. The additional step of NaOH mediated strand cleavage, although possibly enhancing the overall readout (manufactures details), was not included in this assay, as it would not alter the relative results of the experiments. Cleavage reactions were stopped by addition of 20 µl 0.4 % fushin in formamide and denaturation at 90°C for 3 min, followed by quenching on ice. Samples were resolved on 17.5 % TBE-urea gels at 200 V and visualised using a Typhoon scanner for fluorescence imaging (Filter: 526 SP (532 nm), Laser: Blue 488 nm).

**Figure 2:**
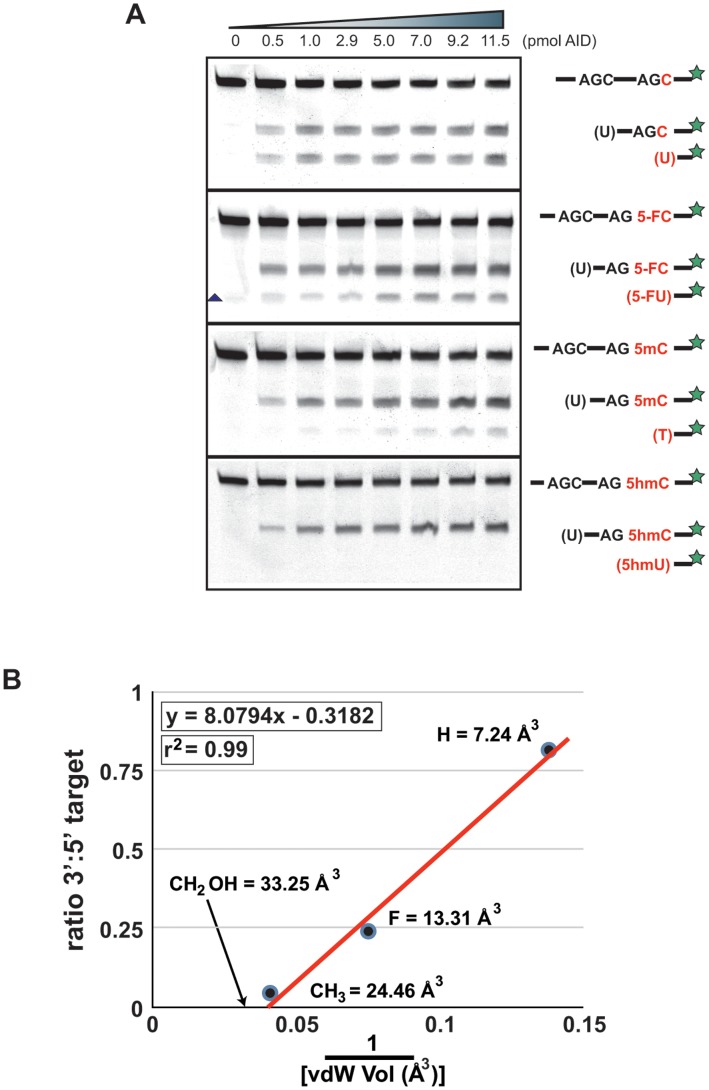
Excess enzyme does not lead to 5 **hmC deamination.** (**A**) The ssDNA oligonucleotide deamination assay was performed for 15 min at 37° C with increasing amounts of enzyme (0–11.5 pmol AID) and analysed as in Figure 1. Labels are as in Figure 1. Triangle indicates a nonspecific cleavage product excluded from quantitation. Each substrate oligo was tested at least 3 times, representative gels are shown. (**B**) The gels were quantitated, and analysed as in Figure 1 F, with the resulting average 3′ target to 5′target ratio plotted against the inverse vdWv.

The van der Waals volumes (vdWv) of the C5 modifications of cytosine were calculated as described [13]. As the oligos contained two AGCs, it was possible that some of the 5**′** AGC targeting was not observed due to a second 3**′** targeting on the same oligo. To this end we calculated the Poisson frequency of a second hit on the same oligo, and added this value to the observed % conversion at the 5**′** AGC.

## Results

Understanding the kinetics of DNA deaminases provides an important aspect of how these proteins function and can be regulated [11]. In a biochemically controlled manner AID possessed activity towards 5 mC [1], initiating the epigenetics field into studying how DNA deaminases can influence DNA methylation marks. It is therefore vital that the current hypothesis on substrate choice for AID is tested by biochemical means rather than idle speculation. In order to test for AID’s competence to deaminate C5-modified cytosines, we generated substrate oligos harbouring modifications that vary within their vdWv of C5-side chains (listed in Figure 1B).

### MtMig can Recognise Modified Deaminated Products

Firstly, we had to prove that the ODA is suitable for using modified substrate oligos. Previously, we monitored AID activity on 5 mC by annealing a complementary oligo to the substrate post-AID incubation [1], followed by incubation with MtMig enzyme for quantitating the deamination. This DNA glycosylase, unlike human TDG or UDG, belongs to the helix-hairpin-helix family of glycosylases [14]. It has been proposed that MtMig recognises mismatches opposite G as strain energy in dsDNA [15]. To test if MtMig can recognise the modified deaminated bases, we synthesised oligos containing the various deaminated products: uracil (U), 5-fluro-uracil (5FU), thymine (T), and 5-hydroxymethyl uracil (5 hmU). After annealing to the second strand oligo with a dG opposite the target base, we incubated the indicated dsDNA with the MtMig enzyme for 30 min at 47°C. As seen in Figure 1A, MtMig was able to efficiently recognize the mismatches, remove the bases, and cleave the backbone, thereby producing fragments that migrated faster through the gel. Neither the absence of MtMig nor un-deaminated modified base (C, 5FC, 5 mC, or 5hmC) resulted in a product. These data indicated that MtMig is capable of recognising AID deaminations of modified cytosine bases.

### AID Deamination of Modified Bases Over Time

To establish kinetic parameters for the AID deamination, we determined the amount of active AID (12–15 %) within our preparations [11], allowing us to precisely control the number of molecules of active AID within each reaction. Furthermore, using various substrate and enzyme concentrations we were unable to establish Michaelis-Menten (MM) like kinetics for AID activity (data not shown). Although we hope that future work will allow us to determine which parameter of the reaction kinetics caused non-MM kinetics, we decided to pursue rate determination without calculating Km, Kd, or Vmax – as this could be misleading.

A time dependent ODA was performed with a near excess of substrate (2.5 pmol oligo and 1.84 pmol AID), demonstrating that recombinant AID was able to deaminate C, 5FC, and 5 mC (Figure 1B). However, 5 hmC could not be converted, even after prolonged incubations. The substrate conversion per enzyme over time was determined from the linear phase (early time points - Figure 1C) of the reactions (pmol product/ pmol enzyme/ min). This activity was plotted against the inverse vdWv for each modified substrate (Figure 1E), this type of graphical conversion allowed for the determination of the theoretical maximum volume (X intercept) AID can accommodate. A line of best fit was applied to the data, which showed a very strong correlation (r^2^ = 0.99). The X intercept was calculated and converted to vdWv, giving a theoretical maximum of 27.28 (±1.05) Å^3^. This volume is below that of 5 hmC (33.25), indicating that AID is unlikely to act on 5 hmC, even theoretically. Aside from determining the activity on the 3′-target, the conversion for the 5′-target (unmodified C) was also measured (Figure 1D). There was no significant difference in deamination, demonstrating that the presence of the various cytosine modifications did not alter the overall kinetics of AID, i.e. there was no indirect effect on AID activity. Since the 3**′** target did not influence the 5**′** target we could use the ratio between the two sites as a measure of AID efficiency. This ratio was plotted against the inverse of the vdWv (Figure 1F), and this approach also demonstrated that the theoretical maximum of C5 (26.14 (±0.20) Å^3^) is below that of 5 hmC.

### Excess AID Enzyme does not Deaminate 5hmC

To determine if the lack of 5hmC deamination was due to working with near substrate excess, we also performed AID deaminations on the oligos using increasing amounts of enzyme. As shown in Figure 2, increasing AID concentration lead to a linear increase in deamination on C, 5FC, and 5 mC substrates (Figure 2A). As with the time dependence, we did not observe any deamination on 5 hmC. Furthermore, neither overexposure of the same gel, nor incubation with 100 pmol of AID (60 fold more than in Figure 2) for 2 h showed any 5 hmU product formation (data not shown). The gels from Figure 2A were quantitated, and the ratio between the 3**′** and 5**′** target calculated and plotted against the inverse of the vdWv (Figure 2B). X intercept calculations showed a theoretical maximum of 25.39 Å^3^. As in the time dependent experiment, this volume is smaller than the volume of the 5 hmC side chain, indicating that 5 hmC is unlikely to be a substrate for AID.

## Discussion

A recent study has suggested a cooperation of TET1 and AID in demethylating DNA duplex [9] unfortunately a direct coupled activity was not demonstrated. However, further modifications of 5 hmC may be a pre-requisite step, since DNA glycosylases such as TDG, SMUG1, and MBD4 were shown to exhibit robust excision activity against 5 hmU:G in dsDNA, but no or very low 5 hmC glycosylase activity *in vitro* [6], [8], [16], [17], and tissue extracts from *smug1* knockout mice lack nearly all hmU-DNA excision activity, suggesting it to be the dominant glycosylase for hmU [17].

Here, using modified C5 carbons of dC (hydrogen, fluoro, CH_3_, or CH_2_OH) we demonstrated a correlation between the inverse size of the C5 side chain vdWv and AID activity, which excluded 5 hmC as a substrate. We cannot fully disregard the shape (as CH_2_OH is more ellipsoid) or electron density at C5 as a possible factor influencing AID activity, but size seems to be the major determinant. This implies that AID is unlikely to be active in the TET dependent 5 mC modification pathway. Importantly, work by Kohli and colleagues [18], also demonstrates that modifications of the C5 side-chain inhibits AID activity and AID was unable to deaminate 5 hmC, supporting our finding. It cannot be fully disregarded that modifications of AID could lead to acceptance of 5 hmC as a substrate, nor that some yet to be identified activity of other DNA deaminases can lead to 5 hmC deamination. These results also have to be placed into an evolutionary context, where AID is the ancestral protein of APOBEC3 and appears prior to the TET family of proteins.

It is therefore more likely that subsequent modifications of 5 hmC (e.g. 5fC and 5caC) will be important in the TET pathway [6], [7]. 5fC and 5caC have been shown to be excised by the above-mentioned TDG [19]. This novel function of TDG may explain its developmental requirement, as TDG knock-out mice are embryonically lethal [20], while UNG [21], SMUG1 [17], and MBD4 [22] deficient mice thrive.

This work is the first to indicate, that there are at least two separate molecular mechanisms that have evolved in counteracting DNA methylation. One of which will go through a base modification that is irreversible and requires removal (AID), while the other will use a modification (TET), which simply ‘hides’ the methyl group from recognition. The later can be processed further to become a substrate for replacement, or it could be reversed back to 5 mC. Because AID induces base lesions that lead to extensive DNA demethylation [2], it will be important to determine to what extent DNA damage and other DNA instabilities will induce local or global DNA demethylation.

## References

[1] Morgan HD, Dean W, Coker HA, Reik W, Petersen-Mahrt SK (2004) Activation-induced cytidine deaminase deaminates 5-methylcytosine in DNA and is expressed in pluripotent tissues: implications for epigenetic reprogramming. The Journal of biological chemistry 279: 52353–52360. 15448152 10.1074/jbc.M407695200

[2] Popp C, Dean W, Feng S, Cokus SJ, Andrews S, et al (2010) Genome-wide erasure of DNA methylation in mouse primordial germ cells is affected by AID deficiency. Nature 463: 1101–1105. 20098412 10.1038/nature08829 PMC2965733

[3] Bhutani N, Brady JJ, Damian M, Sacco A, Corbel SY, et al (2010) Reprogramming towards pluripotency requires AID-dependent DNA demethylation. Nature 463: 1042–1047. 20027182 10.1038/nature08752 PMC2906123

[4] Rai K, Huggins IJ, James SR, Karpf AR, Jones DA, et al (2008) DNA demethylation in zebrafish involves the coupling of a deaminase, a glycosylase, and gadd45. Cell 135: 1201–1212. 19109892 10.1016/j.cell.2008.11.042 PMC2629358

[5] Tahiliani M, Koh KP, Shen Y, Pastor WA, Bandukwala H, et al (2009) Conversion of 5-methylcytosine to 5-hydroxymethylcytosine in mammalian DNA by MLL partner TET1. Science 324: 930–935. 19372391 10.1126/science.1170116 PMC2715015

[6] He YF, Li BZ, Li Z, Liu P, Wang Y, et al (2011) Tet-mediated formation of 5-carboxylcytosine and its excision by TDG in mammalian DNA. Science 333: 1303–1307. 21817016 10.1126/science.1210944 PMC3462231

[7] Inoue A, Shen L, Dai Q, He C, Zhang Y (2011) Generation and replication-dependent dilution of 5fC and 5caC during mouse preimplantation development. Cell research 21: 1670–1676. 22124233 10.1038/cr.2011.189 PMC3357997

[8] Cortellino S, Xu J, Sannai M, Moore R, Caretti E, et al (2011) Thymine DNA glycosylase is essential for active DNA demethylation by linked deamination-base excision repair. Cell 146: 67–79. 21722948 10.1016/j.cell.2011.06.020 PMC3230223

[9] Guo JU, Su Y, Zhong C, Ming G-l, Song H (2011) Hydroxylation of 5-methylcytosine by TET1 promotes active DNA demethylation in the adult brain. Cell 145: 423–434. 21496894 10.1016/j.cell.2011.03.022 PMC3088758

[10] Coker HA, Morgan HD, Petersen-Mahrt SK (2006) Genetic and in vitro assays of DNA deamination. Methods in enzymology 408: 156–170. 16793368 10.1016/S0076-6879(06)08010-4

[11] Coker HA, Petersen-Mahrt SK (2007) The nuclear DNA deaminase AID functions distributively whereas cytoplasmic APOBEC3G has a processive mode of action. DNA repair 6: 235–243. 17161027 10.1016/j.dnarep.2006.10.001

[12] Fersht AR, Ashford JS, Bruton CJ, Jakes R, Koch GL, et al (1975) Active site titration and aminoacyl adenylate binding stoichiometry of aminoacyl-tRNA synthetases. Biochemistry 14: 1–4. 1109585 10.1021/bi00672a001

[13] Zhao YH, Abraham MH, Zissimos AM (2003) Fast calculation of van der Waals volume as a sum of atomic and bond contributions and its application to drug compounds. The Journal of organic chemistry 68: 7368–7373. 12968888 10.1021/jo034808o

[14] Fromme JC, Banerjee A, Verdine GL (2004) DNA glycosylase recognition and catalysis. Current Opinion in Structural Biology 14: 43–49. 15102448 10.1016/j.sbi.2004.01.003

[15] Mol CD, Arvai AS, Begley TJ, Cunningham RP, Tainer JA (2002) Structure and activity of a thermostable thymine-DNA glycosylase: evidence for base twisting to remove mismatched normal DNA bases. Journal of Molecular Biology 315: 373–384. 11786018 10.1006/jmbi.2001.5264

[16] Hashimoto H, Liu Y, Upadhyay AK, Chang Y, Howerton SB, et al (2012) Recognition and potential mechanisms for replication and erasure of cytosine hydroxymethylation. Nucleic acids research. 10.1093/nar/gks155 PMC3367191 22362737

[17] Kemmerich K, Dingler FA, Rada C, Neuberger MS (2012) Germline ablation of SMUG1 DNA glycosylase causes loss of 5-hydroxymethyluracil- and UNG-backup uracil-excision activities and increases cancer predisposition of Ung-/-Msh2-/- mice. Nucleic acids research. 10.1093/nar/gks259 PMC3401444 22447450

[18] Nabel CS, Jia H, Ye Y, Shen L, Goldschmidt HL, et al. (2012) AID/APOBEC deaminases disfavor modified cytosines implicated in DNA demethylation. Nature Chemical Biology in press. 10.1038/nchembio.1042 PMC3427411 22772155

[19] Maiti A, Drohat AC (2011) Thymine DNA glycosylase can rapidly excise 5-formylcytosine and 5-carboxylcytosine: potential implications for active demethylation of CpG sites. J Biol Chem 286: 35334–35338. 21862836 10.1074/jbc.C111.284620 PMC3195571

[20] Cortazar D, Kunz C, Selfridge J, Lettieri T, Saito Y, et al (2011) Embryonic lethal phenotype reveals a function of TDG in maintaining epigenetic stability. Nature 470: 419–423. 21278727 10.1038/nature09672

[21] Rada C, Williams GT, Nilsen H, Barnes DE, Lindahl T, et al (2002) Immunoglobulin isotype switching is inhibited and somatic hypermutation perturbed in UNG-deficient mice. Curr Biol 12: 1748–1755. 12401169 10.1016/s0960-9822(02)01215-0

[22] Millar CB, Guy J, Sansom OJ, Selfridge J, MacDougall E, et al (2002) Enhanced CpG mutability and tumorigenesis in MBD4-deficient mice. Science 297: 403–405. 12130785 10.1126/science.1073354

